# Prevalence of severe cardiovascular abnormalities amongst West African HIV-infected patients on antiretroviral therapy followed at a referral HIV centre

**DOI:** 10.4102/sajid.v36i1.187

**Published:** 2021-02-16

**Authors:** Frederic N. Ello, Esaie K. Soya, N’douba A. Kassi, Patrick A. Coffie, Gisèle A. Koaukou, Melaine C. Mossou, Doumbia Adama, Icklo Coulibaly, Eboi Ehui, Aristophane Tanon, Serge P. Eholie

**Affiliations:** 1 Département de Dermatologie-Infectiologie, Unité de Formation et de Recherche des Sciences Médicales, Université Felix Houphouët-Boigny, Abidjan, Côte d’Ivoire; 2 Service des Maladies Infectieuses et Tropicales, Centre Hospitalier Universitaire de Treichville, Abidjan, Côte d’Ivoire; 3 Institut de Cardiologie d’Abidjan, Abidjan, Côte d’Ivoire

**Keywords:** prevalence, cardiovascular disease, HIV, antiretroviral treatment, CD4 count

## Abstract

**Background:**

With success and effective long-term antiretroviral treatment (ART), HIV-infected patients live longer and frequently developed non-communicable diseases (NCDs). Few studies have been conducted in low-income countries, particularly in West Africa.

**Methods:**

We carried out a cross-sectional study in the referral HIV centre of the Service des Maladies Infectieuses et Tropicales (SMIT) in Abidjan. From April to September 2015, we consecutively included HIV-1 infected patients aged 18 years and older, and on ART for a minimum of 12 months. Data were collected using a structured questionnaire, and entered into the centre’s computerised HIV database. Clinical assessment, laboratory tests, electrocardiogram, transthoracic echocardiography and vascular Doppler ultrasound were performed. The main outcome was the prevalence of patients with severe cardiovascular abnormalities (SCA). Univariate and multivariate logistic regressions were used to identify factors associated with SCA.

**Results:**

Out of 278 patients (median age 46 years, interquartile range [IQR: 41–52]), 74.5% were female. Overall, the median duration of ART was 84 months (IQR: 54–126). One hundred and ninety-nine (71.6%) patients were on first-line ART regimen and 229 (82.4%) were virologically suppressed with a median CD4 count of 511 cells/mm^3^ (IQR: 347–529). Basically, cardiovascular abnormalities were mainly non-obstructive carotid plaques (19.1%) followed with left ventricular diastolic dysfunction (16.5%). The overall prevalence of SCA in the study population was 7.6% (95% Confidence Interval [95% CI]: 4.7–11.3). The prevalence of SCA 7.6% (95% Confidence Interval [95% CI]: 4.7–11.3). In multivariate analysis, age > 50 years and nadir CD4 count > 200 cells/mm^3^ were significant predictors of SCA.

**Conclusion:**

The prevalence of SCA is high in West African HIV-treated patients. Given the high mortality associated with cardiovascular diseases in the general population, refining disease preventive strategies in HIV-positive subjects is essential to continue prolonging their life.

## Introduction

Antiretroviral treatment (ART) has dramatically reduced AIDS-related morbidity and mortality.^1,2,3^ As a result, the increase in life expectancy of HIV-infected patients has been associated with the occurrence of chronic, late HIV-related complications and non–communicable diseases (NCDs) such as cancers, neurocognitive disorders, metabolic disorders, diabetes and cardiovascular diseases (CVD). These are significant causes of severe morbidity and mortality observed in HIV-positive individuals as compared to the general population.^4,5,6^ Apart from the traditional risk factors including advanced age, male sex, family history of CVD, higher smoking rates and dyslipidemia, individuals may also develop CVD because of non-traditional factors such as inflammation, the direct effects of the virus on the vasculature and the toxicity of specific antiretroviral drugs, causing metabolic syndrome and insulin resistance.^7,8^

In developed countries 9% – 20% of HIV-positive patients have a moderate to high risk of myocardial infarction over a 10-year period, identified as the main CVD in these countries with smoking as the main factor.^9,10^ With the changes in lifestyle and the increasing number of people living in urban cities, CVD are becoming an increasing public health issue in low-income countries heavily affected by HIV and AIDS where unfortunately, few data are available of patients on ART. Most previous studies were carried out in Eastern and Southern Africa (Sub-Saharan Africa Survey of Heart Failure and Soweto study cohort), and reported a significantly lower rate of CVD in HIV-infected individuals compared to industrialised countries.^11,12^ Eholie et al. estimated the 10-year cardiovascular risk at 3% using Framingham score, during follow-up of HIV-infected patients on ART with no difference amongst sub-Saharan patients living in Côte d’Ivoire or France.^13^ However, the patterns of CVD in HIV-treated patients have not yet been documented in Côte d’Ivoire. Our study aimed to estimate the prevalence of severe cardiovascular abnormalities (SCA) as measured by electrocardiogram, echocardiography, vascular Doppler in patients on ART and to assess associated factors which could result in public health interventions so as to reduce these non-AIDS events in people living with HIV (PLHIV).

## Methods

### Study setting, population and recruitment

A cross-sectional study was conducted in the Service des Maladies Infectieuses et Tropicales (SMIT) of CHU Treichville Hospital in Abidjan from April to September 2015. The SMIT is a referral HIV center for the management of HIV-positive individuals, working closely with several others institutions in Africa and Europe. On the whole, 16 906 HIV-infected patients were in care in the SMIT and 9881 received ART. Moreover, 3575 patients had been on ART regimen for at least 12 months. All ART-naive patients started first-line ART with a WHO-recommended regimen containing at least two nucleoside reverse transcriptase inhibitors (NRTIs) and one non-nucleoside reverse transcriptase inhibitor (NNRTI).^14^ At the time of our study, the ART-start CD4 threshold was 350 cells/mm^3^ in asymptomatic HIV-infected adults.^14^ Second-line ART for adults consisted of two nucleoside reverse-transcriptase inhibitors (NRTIs) and a ritonavir-boosted protease inhibitor (PI). Atazanvir/ritonavir (ATV/r) and Lopinavir/ritonavir (LPV/r) heat-stable fixed-dose combinations are the preferred boosted PI options for second-line ART. Antiretroviral treatment and biological examinations such as CD4 cell count, haematology and biochemistry were provided free of charge by the national HIV/AIDS control programmes according to their individual care package. Lipid and cardiovascular assessment were not subsidised. We included in this analysis HIV-1 infected patients aged 18 years or older, treated for at least 12 months.

Exclusion criteria were: HIV-2 infected or HIV-1/2 dually reactive patients, ART-naïve patients, and patients with any acute infectious episode.

### Clinical, laboratory examinations and cardiac measurements

Demographic data, self-reported walking time, cigarette smoking status, alcohol intake and the history of ART was collected at inclusion. The self-reported walking time was categorised into two modalities: greater than 30 min per day or less than 30 min per day. Alcohol consumption was assessed using the Alcohol Use Disorders Identification Test (AUDIT).^15^ Smoking was categorised into never smokers, current smokers or former smokers using the Natural Language Processing (NLP) tools.^16^ The standardised examination consisted of a targeted assessment of medical history and a physical examination including two separate measures of blood pressure and anthropometrics measurements (height, weight, waist and hip circumference). We performed laboratory analyses (haematology, serum chemistries and CD4 T-cell counts), HIV-1 RNA viral load testing (COBAS Amplicor HIV-1 Monitor Test, version 1.5, with a lower limit detection of 50 copies per milliliter).

The cardiac examination was performed by the same cardiologist. A resting 12-lead electrocardiogram (Schiller A-T 110 machine) was used to diagnose conduction disorders and rhythm abnormalities.

Electrocardiography abnormalities were classified using the Minnesota ECG Code classification system.^17^

A 2-dimensional echocardiography (Vivid S6 machine) was used to assess the left ventricular mass and ejection fraction and evaluate the cavity size and valves of the heart. The m-mode measurements of left ventricular (LV) and left atrial dimensions were obtained in the parasternal long-axis view. Left ventricular mass was calculated according to Devereux et al.’s formula and normalised to body surface area and height^2,7^ to obtain LVM index (LVMI).^18^ Left ventricular ejection fraction was assessed using Simpson’s biplane rule using conventional apical 4- and 2-chamber views.^18,19^

Vascular Doppler (Vivid S6 ultrasound machine) equipped with a 7 MHz linear probe with high-axial resolution was used to measure carotid intima media thickness (cIMT) and determine the plaque presence on the left and right carotid bifurcations and internal and common carotid arteries. Carotid artery plaque was defined as a focal structure that encroaches into the arterial lumen of at least 0.5 mm or 50% of the close IMT value or demonstrates a thickness > 1.5 mm, as measured from the media-adventitia interface to the intima-lumen interface.^20^

### Study outcomes

All cardiovascular abnormalities observed after CVD examinations in patients were reported. Based on the 10th review of the International Statistical Classification and Related Health Problems (ICD-10), the cardiovascular abnormality meet the definitions of ‘severe’ if it requires a cardiology consultation, with cardiovascular medication and requires hospitalisation or endangers the life of the patient (this corresponds to an event during which the participant is at real risk of death).^21^

In the electrocardiogram LV Hypertrophy was defined using Cornell index (RaVL + SV3) > 28 mm in men and 20 mm in women. Repolarisation disorders were found corresponding to ST segment elevation and ST-T wave changes. Left ventricular (LV) dilation was defined as indexed LV diameter in diastole > 34 mm/m^2^, and left atrial (LA) enlargement as LA diameter > 2.6 mm/m^2^. Left ventricular hypertrophy (LVH) was defined as an indexed LV mass (LVMI) > 131 g/m^2^ in men and LVMI > 108 g/m^2^ in women. We used Appleton’s criteria to diagnose and classify LV diastolic dysfunction.^22^

Dilated cardiomyopathy (DCM) is defined as left ventricular (LV) dilation and systolic dysfunction in the absence of coronary artery disease or abnormal loading conditions proportionate to the degree of LV impairment.^23^

The diagnostic of pulmonary hypertension (PH) is based on a mean pulmonary artery pressure of more than 25 mm Hg at rest, or more than 30 mm Hg with exercise, measured by Doppler echocardiography.^24^

Carotid artery stenosis is a narrowing or constriction of any part of the carotid arteries. Any stenosis greater than 50% was considered significant according to the San Francisco consensus conference.^25^

Subclinical atherosclerosis was defined as cIMT ≥ 0.9 mm and/or the presence of ≥ 1 carotid plaque.

Thromboses were the formation of a blood clot inside a blood vessel, obstructing the flow of blood through the circulatory system. Thrombosis may occur in veins (venous thrombosis) or in arteries (arterial thrombosis).

### Statistical analysis

Calculation of the sample size was based on an expected 20% prevalence of CVD in the study population. Therefore, the inclusion of 245 patients would have led to at least 49 cases of CVD (*α* risk of 0.05 and 1-*β* power of 80%). To account for patients who could refuse the survey (10%), a total of 270 HIV-infected patients on ART were required in the present study. Categorical variables were described with numbers and percentages, and continuous variables with median and interquartile range (IQR). The prevalence of SCA was calculated as the number of patients meeting the definition out of the total population with their corresponding 95% confidence interval (95% CI). We used a univariable analysis and then a multivariable logistic regression analysis to identify factors associated with SCA. Statistical analysis was performed using Stata statistical software for professionals (Stata^TM^ 10.0; Stata Corporation, College Station, TX, USA).

### Ethical considerations

This study complies with ethical standards as set out in the Declaration of Helsinki. It received approval from the Ivory Coast National Ethics Committee for Health Research. All patients gave written, informed consent for participating in the study. Illiterate patients were required to have an independent witness with them during the information session. After obtaining patients’ verbal consent for their study participation, the participant was required to sign and date the informed consent form, witnessed by the research physician.

## Results

### Baseline characteristics

The median age was 46 years (IQR: 41–52) and the majority (207, 74.5%) of them were women. Most patients (177, 63.7%) were not sedentary and reported walking more than 30 min per day. Frequent alcohol consumption was reported by 110 (40%) patients and few of them 20 (7.2%) were regular smokers. Eleven (4%) patients had a history of diabetes mellitus. Eighty-six and 36 patients had hypertension and were obese (BMI ≥ 30 kg/m^2^), respectively. The majority of patients were asymptomatic. Only 10 (3.6%) patients reported exertional dyspnea. One hundred and seventy-two (61.9%) participants were on two nucleoside reverse transcriptase inhibitors and one non-nucleoside reverse transcriptase inhibitors regimens (2NRTI + 1NNRTI). Seventy-nine (28.4%) patients were on 2NRTI and one protease inhibitor regimens (2NRTI + 1 PI). The median duration of ART was 84 months (IQR, 54–126 months) at the time of evaluation. Additional characteristics are shown in Table 1.

**TABLE 1:** Baseline characteristics and prevalence of severe cardiovascular abnormalities, Abidjan, April 2015 – September 2015 (*N* = 278).

Characteristics	*n*	%	Median	IQR
**Socio-demographics indicators**				
Age (years)			46	41–52
**Gender**			-	-
Male	71	25.5	-	-
Female	207	74.5	-	-
**Lifestyle**			-	-
Walking time (> 30 min per day)	177	63.7	-	-
Current or former smoker	20	7.2	-	-
Alcohol consumption	110	40.0	-	-
**Cardiovascular risk factors**				
Body mass index (kg/m^2^)				
Obese (≥ 30)	36	13	-	-
Overweight (25–30)	101	36.3	-	-
Previous cardiovascular disease	10	3.6	-	-
Diabetes mellitus	11	4	-	-
Blood pressure (≥ 140/90 mmHg)	86	30.9	-	-
**HIV disease characteristics**				
HIV CDC classification stage C	119	42.8	-	-
**CD4 + lymphocytes (cells/mm^3^)**				
Current	-	-	511	347–529
Nadir	-	-	234	104–251
**HIV-RNA (copies/ml)**				
> 1000	36	13	-	-
50–1000	13	4.6	-	-
≤ 50	229	82.4	-	-
**Antiretroviral treatment**				
Median duration of ART (months)	-	-	84	54–126
**Type of ART regimen**				
2 NRTIs + 1 PI	79	28.4	-	-
3 NRTIs	27	9.7	-	-
2 NRTIs + 1 NNRTI	172	61.9	-	-
**Laboratory evaluation**				
Blood glucose levels (g/l)	-	-	0.87	0.8–0.96
ALT levels (IU/l)	-	-	20	15.5–29
Total cholesterol. g/l	-	-	2.11	1.76–2.41
HDL-c. g/l	-	-	0.61	0.5–0.76
LDL-c. g/l	-	-	1.19	0.92–1.51
TG. g/l	-	-	1.03	0.79–1.44
**Cardiovascular abnormalities**				
ECG abnormalities†	70	25.2	-	-
Echographic abnormalities‡	95	34.2	-	-
Subclinical atherosclerosis§	59	21.2	-	-
**Number of abnormalities by patient**				
≥ 1	159	57.0	-	-
1	99	35.6	-	-
2	55	19.8	-	-
3	5	1.8	-	-
**Severe cardiovascular abnormalities**				
Pulmonary hypertension	12	4.4	-	-
Dilated cardiomyopathy	3	1.1	-	-
Obstructive carotid plaques	3	1.1	-	-
Deep venous thrombosis	2	0.7	-	-
Arteriopathy of the lower limbs	1	0.4	-	-
Global of SCA¶	21	7.6	-	-

SCA, Severe cardiovascular abnormalities; HIV CDC, HIV Center of Diseases Control; HDL, High-density lipoprotein; IQR, interquartile range; LDL, Low-density lipoprotein; NNRTI, Non-nucleoside reverse-transcriptase inhibitor; NRTI, Nucleoside reverse-transcriptase inhibitor; PI, Protease inhibitor; TG, Triglyceride, ALT, Alanine Transferase.

† , Left ventricular hypertrophy:22 (7.9%); Left atrial dilatation: 7 (2.5%); Repolarisation disorders: 28 (10.1%); Short PR interval: 01 (0.4%); Conductions disorders: 04 (1.4%); Microvoltage: 02 (0.7%); Arrhythmias: 06 (2.2%);

‡ , Left ventricular diastolic dysfunction: 46 (16.5%); Left ventricular hypertrophy: 03 (1.1%); Left atrial enlargement: 17 (6.1%); Dilated cardiomyopathy:03 (1.1%); Pulmonary hypertension:12 (4.4%); Valvular insufficiency: 10 (3.6%), Pericardial effusion: 04 (1.4%);

§ , Non-obstructive carotid plaques: 53 (19.1%); Obstructive carotid plaques: 03 (1.1); Deep venous thrombosis: 02 (0.7%); Arteriopathy of the lower limbs: 01 (1.1%);

¶ , 95% CI = 4.7–11.3.

### Prevalence of severe cardiovascular abnormalities

On the whole, cardiovascular disorders were found in 159 (57%) patients, 32% of whom were elderly (over 50 years) patients. Electrocardiography abnormalities were observed in 70 (25.2 %) patients included. Repolarisation disorders and left ventricular hypertrophy were the most common reported (10.1% and 7.9%, respectively). Echocardiography abnormalities were diagnosed in 95 (34.2%) patients, half of whom were elderly patients. Left ventricular diastolic dysfunction was the most frequent abnormality observed on echocardiography in 46 (16.5 %) participants. Subclinical atherosclerosis was found in 59 (21%) patients with predominance of non-obstructive carotid plaques. Severe cardiovascular abnormalities was prevalent in 21 (7.6% [95% CI: 4.7–11.3]) patients distributed as follows: dilated cardiomyopathy, *n* = 3; pulmonary hypertension, *n* = 12; obstructive carotid plaques, *n* = 3; deep venous thrombosis, *n* = 2; arteriopathy of the lower limbs, *n* = 1. The overall cardiovascular abnormalities are presented in Table 1.

### Factors associated with severe cardiovascular abnormalities

Table 2 shows the results of the SCA univariate analysis. In univariate analysis, factors significantly associated with the presence of SCA were: age > 50 years, participants walking time per day *˂* 30 min, hypertension (≥ 140/90 mmHg), ALT levels. > 40 UI, blood glucose levels > 1.1 g/l, and nadir CD4 cell count > 200 cells/mm^3^. Nevertheless, there was no significant association between gender, smoking status, BMI, PI-boosted ART, total cholesterol/high density lipoprotein ratio and viral load.

**TABLE 2:** Results of univariate and multivariate analysis between baseline characteristics and severe cardiovascular abnormalities, Abidjan, April 2015 – September 2015 (*N* = 278).

Variables	Unit	Univariate analysis			Multivariate analysis		
		OR	95*%* CI	*p*-value	aOR	95*%* CI	*p*-value
Age (years)	> 50 vs ≤ 50	2.80	1.14–7.1	0.026	2.92	1.14–7.79	0.027
Gender	Male/Female	1.18	0.41–3.04	0.74	-	-	-
Walking time	≤ 30 min by day/> 30 min by day	1.91	0.72–5.98	0.22	2.47	0.87–8.29	0.10
Smoking status	Current or former smoker/Never smoker	1.40	0.21–5.36	0.67	-	-	-
BMI (Kg/m²)	> 30 vs < 25	1.83	0.44–6.02	0.64* 44	-	-	-
	25 – 30 vs < 25	1.26	0.46–3.42	-	-	-	-
Blood pressure (≥ 140/90 mmHg)	Yes/No	1.87	0.75–4.61	0.17	-	-	-
PI-boosted ART	Yes/No	0.78	0.25–2.08	0.64	-	-	-
ALT levels (IU/l)	> 40 vs ≤ 40	2.69	0.59–9.13	0.14	3.08	0.64–11.23	0.11
Blood glucose levels (g/l)	> 1.1 vs ≤ 1.1	2.94	0.79–8.96	0.07	3.27	0.82–11.14	0.06
TC/HDL ratio	> 4.4 vs ≤ 4.4	0.6	0.14–1.85	0.42	-	-	-
Nadir CD4 count (/mm^3^)	> 200 vs ≤ 200	0.33	0.12–0.84	0.023	0.33	0.12–0.85	0.025
Viral load (copies/ml)	> 1000 vs ≤ 50	1.17	0.25–3.61	0.98*	-	-	-
	50–1000 vs ≤ 50	1.04	0.06–5.78	-	-	-	-

BMI, Body Mass Index; ARV, Antiretroviral; ALT, Alanine Aminotransferase; TC, Total Cholesterol; HDL, High Density Level; OR, Odds Ratio; 95% CI, 95% Confidence Interval; aOR, adjusted Odds Ratio.

* , *P*-value for the group.

In a multivariable analysis, there was a significant association between SCA, the age and nadir CD4 cell count after adjusting the other variables (walking time, ALT levels and blood glucose levels). Indeed, patients aged > 50 years were more likely to present SCA than those aged ≤ 50 years (aOR 2.92; 95% CI 1.14–7.79). Furthermore, patients with nadir CD4 cell count > 200 cells/mm^3^ were (aOR 0.33; 95% CI 0.12–0.85) less likely to present SCE than those with nadir CD4 cell count ≤ 200 cells/mm^3^ (Table 2).

## Discussion

Our main findings showed an estimated 7.6% prevalence of SCA. They were dominated by pulmonary hypertension (PH), dilated cardiomyopathy and obstructive carotid plaques. These exposed patients to life-threatening events such as myocardial infarction, cerebral stroke and decompensated heart failure, if they are not diagnosed and treated on time.^26,27,28^ The prevalence of PH in our study is consistent with a recent narrative review indicating a PH prevalence between 5% and 13% in the same population.^29^ Besides this review, Bigna *et al.* in a systematic review found a pooled prevalence of 14% (95% CI 6%–23%) of PH amongst African HIV-infected adults between 2006 and 2014 from the three African WHO regions.^30^ This is significantly higher than the estimated 0.5% prevalence of HIV-associated pulmonary hypertension in developed countries.^31^ With regard to dilated cardiomyopathy (DCM), we found low rates (1%) of these events which are confirmed by a recent study on HIV-infected children and adolescents with high intake of ART in Uganda.^32^ Indeed, Patel et al. showed a significant impact of ART marked by a 50% reduction of DCM in developing countries. This may explain the low rate observed in our study because more than two-thirds of our patients had full viral suppression.^33^

Advanced age and nadir CD4 cell count were the main factors associated with SCA. In a prospective, multicentre cohort study, Esser et al. reported an estimated 10.1% prevalence of a broad range of CVD in HIV-positives subjects estimated at 10.1%. In this report, aging HIV-positive patients (≥ 45 years, *N* = 348) showed significantly increased rates of CVD. These results are similar to ours except that CVDs are mainly represented by coronary artery diseases and myocardial infarction.^34^

Even the design of our study does not allow us to clearly establish the causal link of the level of CD nadir and cardiovascular anomalies, some data in the literature have also reported it. A case-control study from France noted that, independent of cardiovascular risk factors and ART, HIV replication, a low CD4 T-cell nadir and a high current CD8 T-cell count are associated with an increased risk of MI in HIV-infected individuals.^35^ Data from the SMART study, in combination with a handful of non-randomised studies noting relationships between lower nadir CD4 and increased risk of preclinical or clinical CVDs.^35,36,37,38^ Rasmussen et al. identified an inverse relationship between CD4 nadir and stroke risk, reporting that a nadir CD4 count below 200 cells/µL more than doubled the risk of stroke.^39^ We need to rethink prevention strategies with a minimum systematic non-invasive screening in this population. Finally, the management of these SCA was carried out in the Cardiac Institute of Abidjan and adjusted according to the types of events.

Our study has several limitations. The cross-sectional design restricts any causal inference. The relatively small sample size of the study (*N* = 278) certainly resulted in a loss of power and precision in the analysis. Finally, the absence of control groups (e.g. HIV-naive subjects) limits the validity of the results. Carefully collected epidemiologic data comparing HIV-positive to HIV-negative patients in low- and middle-income countries are critical to improve understanding. Ongoing longitudinal studies are necessary to determine whether our findings have any significant impact on future heart function and the real contribution that treatment-related factors have on progression, incidence and prevention of cardiac disease.

## Conclusion

The prevalence of SCA is high in West African HIV-treated patients. Therefore, a standardised screening and risk reduction intervention should be routinely undertaken amongst elderly HIV-infected patients receiving ART. In view of the importance of this issue, it appears essential to conduct longitudinal studies to further evaluate the impact of HAART on CVD in the sub-Saharan region and beyond.
